# First Molecular Identification of *Taenia hydatigena* in Wild Ungulates in Poland

**DOI:** 10.1007/s10393-019-01392-9

**Published:** 2019-01-23

**Authors:** Katarzyna Justyna Filip, Anna Maria Pyziel, Witold Jeżewski, Anna Weronika Myczka, Aleksander Wiaczesław Demiaszkiewicz, Zdzisław Laskowski

**Affiliations:** 1 W. Stefański Institute of Parasitology PAS, Twarda 51/55, 00-818 Warsaw, Poland; 2 Department of Food Hygiene and Public Health Protection, Faculty of Veterinary Medicine, Warsaw University of Life Sciences-SGGW, Nowoursynowska 166, 02-787 Warsaw, Poland

**Keywords:** *Taenia hydatigena*, Cysticercosis, Moose, Wild boar, Wolf

## Abstract

The article examines the presence of metacestodes on the liver capsule in two wild boars and on the liver capsule and mediastinum in moose. Cysticerci were identified as *Taenia hydatigena* metacestodes by morphological features—size and shape of rostellar hooks and molecular analysis of the partial sequences of cytochrome c oxidase subunit 1 gene. Molecular analysis revealed similarities between the metacestodes isolated from the wild mammals in the present study to *T. hydatigena* in European wolves. It is possible that infection of moose and wild boar with *T. hydatigena* metacestode is related to the growth in the wolf population in Europe.

**Electronic supplementary material:**

The online version of this article (10.1007/s10393-019-01392-9) contains supplementary material, which is available to authorized users.

## Introduction

*Taenia hydatigena* is a cosmopolitan tapeworm from domestic and wild canids (Bowman 2009), which can be transmitted to a wide variety of intermediate hosts, including domestic goats, sheep, swine and certain wild ungulates (Bowman 2009; Nguyen et al. 2016). Immediately after migrating through the liver of the intermediate host, the larvae or metacestode of *T. hydatigena*, commonly known as *Cysticercus tenuicollis*, typically encyst on the omentum, mesentery and the serosal surface of the liver; however, they can also be found in the lungs, heart, uterus or kidneys (Gomez-Puerta et al. 2015). Subsequent migration of the larvae can result in traumatic hepatitis, resulting in the death of the host animal (Rostami et al. 2013). Cysticercosis by *T. hydatigena* results in serious economic losses in livestock production due to condemnation of livers or whole carcass at slaughter, which makes it a matter of veterinary importance (Scala et al. 2015).

In Europe, an important reservoir is maintained in the environment for *T. hydatigena* by wolves and dogs, which act as definitive hosts, and wild ungulates, which act as intermediate hosts (Gori et al. 2015; Otranto et al. 2015; Lesniak et al. 2017). *Taenia hydatigena* metacestodes were found in 12.3% of game cervids in Slovakia (Letkova et al. 2008), 22% of examined wild ruminants in Belarussian Polesie (Shimalov and Shimalov 2003) and 20% of wild boars from Saarema in Western Estonia (Järvis et al. 2007).

In Poland, little epidemiological data regarding the occurrence of *T. hydatigena* is available and all reports refer exclusively to the infection of intermediate hosts. In the 1960s, cysticercosis was reported in all species of Polish cervidae (Dróżdż 1966). Since then, *T. hydatigena* metacestode has been found in a single wild boar, and a number of roe deer and moose from central Poland (Gadomska 1981; Tropiło and Kiszczak 1995; Filip and Demiaszkiewicz 2016), as well as a few pigs from the north-western part of the country (Kędra et al. 2001).

The aim of this study is to provide a morphological and molecular description of *T. hydatigena* metacestodes identified in wild mammals in Poland.

Twenty wild boars from Strzalowo Forest District (53°45′57.03″N, 21°25′17.79″E), hunted during 2014, were necropsied. A single specimen of metacestode of *T. hydatigena* was found on the liver capsule in two of the boars.

In 2016 and 2017, six moose killed in car accidents in Kampinos National Park and 3 moose in Polesie National Park were necropsied. Two *T. hydatigena* metacestodes were found on the liver capsule and mediastinum of a two-year-old female moose from Dąbrówka Forest District (52°21′03″N 20°36′05″E), Kampinos National Park.

To perform morphological analysis, the intact cysticerci and rostellar hooks of metacestodes were measured. To localize the crowns, the cysticerci with all introverted scoleces were mounted in Faure’s fluid, without staining. The crowns were extracted from the cysticerci and temporarily mounted for photography and study. For morphological identification, the rostellar hooks were liberated from the crowns using an aqueous solution of 1% pepsin and 0.4% HCl and then mounted in Faure’s fluid. Only hooks aligned well in the horizontal plane were used for morphometric analysis and photography. The rostellar hooks were examined according to Haukisalmi et al. (2011), using an Olympus BX50 light microscope with a Cell D digital image analysis system.

The DNA was isolated using the DNA Mikro Kit (Syngen) according to the manufacturer’s protocol. The primers Thg452F (5′-TGCATTTAGCTGGTGCGTCAAGTA -3′) and Thg1326R (5′-ACAAACACGCCGGGGTAACC-3′) were used to partially amplify the mitochondrial cytochrome c oxidase subunit 1 gene (*cox*1). PCRs were conducted in a 50 µl reaction mixture containing 2.0 µl of DNA template, 0.2 µl (1.0U) of HiFiTaq Polymerase (Novazym), 1 µl of dNTPs mix (10 mM), 1.0 µl of each primer (20 mM) and 5 µl of 10 × Polymerase buffer (pH 8.6, 25 mM MgCl2) and 40.8 µl of deionized water. In the negative control, nuclease-free water was added to the PCR mix instead of the tested DNA.

DNA amplification was performed using the DNA Engine T100 Thermal Cycler (Bio-Rad) using the following programs: The initial denaturation was performed at 95°C for one minute, followed by 35 cycles of denaturation at 95°C for 20 s, annealing at 56°C for 20 s and extension at 72°C for 40 s, with a final extension performed at 72°C for five minutes.

The PCR products were visualized on a 1.0% agarose gel (Promega) stained with ethidium bromide. Visualization was performed using ChemiDoc, MP Lab software (Imagine, Bio-Rad). The PCR amplicons were purified using a PCR Clean-Up Kit (Genoplast). The purified PCR products were sequenced in both directions by Genomed (Poland) and assembled into contigs using ContigExpress, Vector NTI Advance 11.0 (Invitrogen Life Technologies, USA). The derived sequences were submitted to GenBank/EMBL.

All isolated metacestodes have been identified as cysticerci of *T. hydatigena* on the basis of size and shape of rostellar hooks and molecular analysis of the partial sequences of cytochrome c oxidase subunit 1 gene.

**Figure 1:**
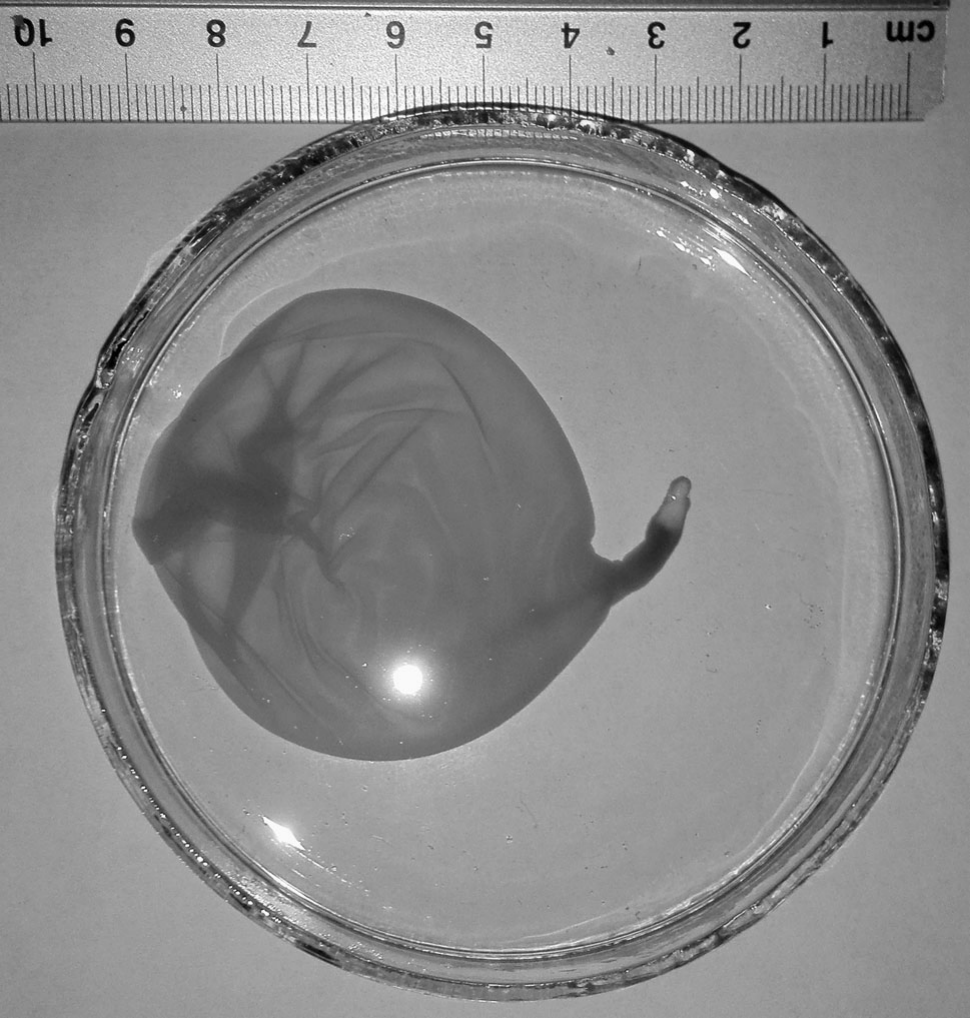
Metacestode of *Taenia hydatigena* from the mediastinum of moose (*Alces alces*).

Cysticerci were oval in shape, opalescent white, with invaginated scolex. Diameter of the metacestodes ranged from 1.5 cm (cysticerci from the liver of wild boars and moose) to 5 cm (cysticerci from the moose mediastinum) (Fig. 1).

**Figure 2:**
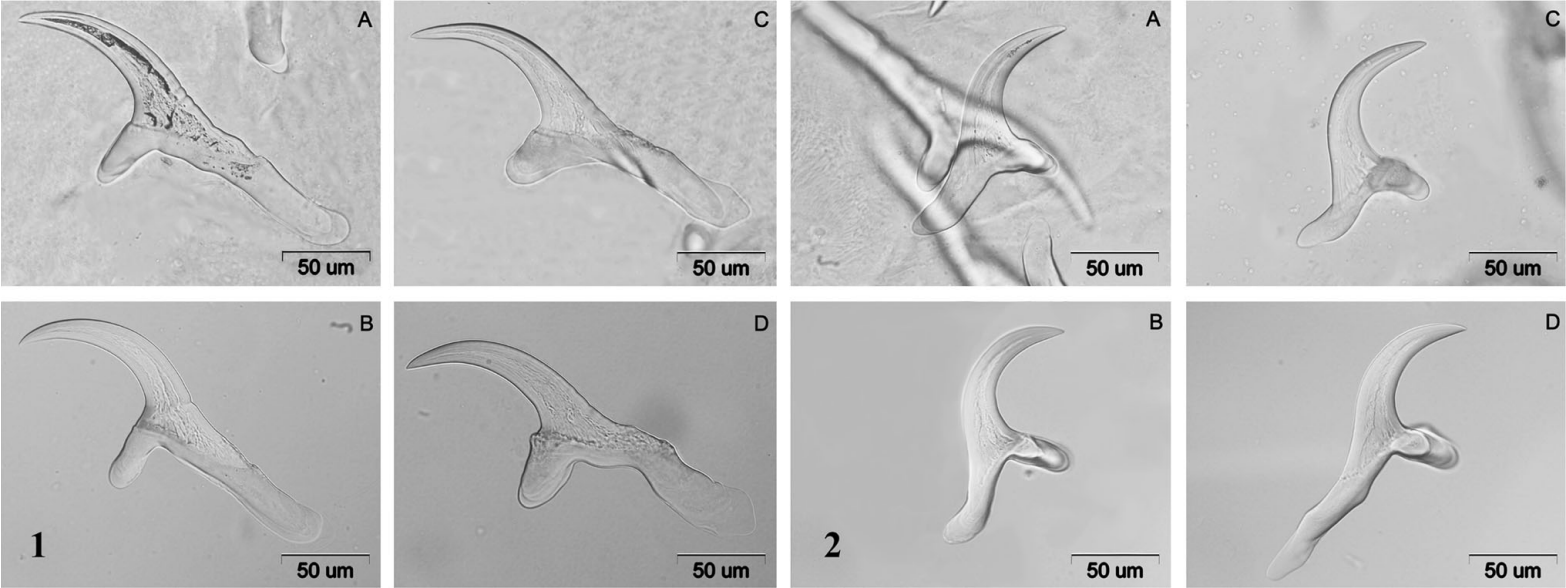
Large (1) and small (2) rostellar hooks of *Taenia hydatigena* metacestode from the liver of wild boars (A, B), liver of moose (C) and mediastinum of moose (D).

The rostellum of *T. hydatigena* metacestodes from wild boars was armed with 32 small and large hooks, while those obtained from the liver and mediastinum in moose had 34 and 30 hooks, respectively (Fig. 2). The measurements of small and large hooks are included in Table 1.

**Table 1:** Measurements of Large and Small Hooks of Polish Isolates of *Taenia hydatigena* Found in the Liver of Two Wild Boars and One Moose (Larva no. 1 Isolated from the Liver; Larva no. 2 Isolated from the Mediastinum).

Feature	*Sus scrofa*						*Alces alces*					
	Individual no.1			Individual no. 2			Larva no. 1			Larva no. 2		
	*n*	Mean	Range	*n*	Mean	Range	*n*	Mean	Range	*n*	Mean	Range
*Large hooks*												
Total length (TL)	6	221.3	219.4–223.7	10	204.9	202.7–206.9	6	225.7	223.5–227.4	7	222.5	218.0–226.0
Total width (TW)	6	74.6	72.6–75.7	10	82.6	80.6–84.4	6	81.5	79.8–83.7	7	78.0	72.4–80.8
Basal length (BL)	6	136.3	132.0–139.4	10	123.2	119.3–128.8	6	146.5	144.6–148.7	7	136.1	131.4–142.0
Apical length (AL)	6	103.1	100.4–108.6	10	98.7	97.3–100.7	6	106.2	103.9–107.8	7	103.4	99.5–106.5
Guard length (GL)	6	36.4	31.9–39.0	10	34.0	32.6–36.9	6	35.1	33.6–36.7	7	34.6	31.7–37.9
Guard width (GW)	6	28.6	24.4–36.9	10	19.2	17.5–20.9	6	21.2	20.5–22.3	7	27.5	24.5–29.3
Blade curvature (BC)	6	19.3	18.3–21.3	10	25.5	24.8–26.5	6	18.5	17.0–19.2	7	19.8	17.8–22.3
Handle width (HW)	6	28.4	24.0–32.8	10	23.3	20.9–26.8	6	22.5	21.2–24.1	7	26.1	24.2–29.7
*Small hooks*												
Total length (TL)	5	131.8	126.8–136.6	11	133.6	131.1–136.3	6	146.8	142.2–149.7	5	171.1	166.3–176
Total width (TW)	5	74.3	69.7–76.5	11	71.8	68.6–79.1	6	72.2	70.2–74.1	5	74.5	72.6–77.5
Basal length (BL)	5	77.7	75.1–80.0	11	79.2	77.3–84.2	6	90.2	87.0–91.9	5	114.4	107.4–123.4
Apical length (AL)	5	85.2	80.0–89.3	11	74.4	70.4–79.4	6	82.0	79.9–84.2	5	80.1	75.4–83.5
Guard length (GL)	5	30.7	25.3–37.3	11	30.8	25.7–37.6	6	26.1	24.5–28.3	5	33.9	30.3–37.3
Guard width (GW)	5	18.6	17.0–20.3	11	15.9	15.3–18.4	6	22.1	20.6–23.4	5	17.7	17.4–18.1
Blade curvature (BC)	5	22.1	20.2–23.6	11	23.1	21.9–23.7	6	19.6	18.1–21.8	5	18.6	16.9–20
Handle width (HW)	5	15.6	14.4–17.2	11	15.3	14.5–16.3	6	18.5	17.9–18.9	5	16.9	15.7–18.4

Four nucleotide sequences of the *cox*1 region of the isolated metacestodes were obtained during the study (GenBank accession nos.: MF630923, MF630924, MF630925, MF630926). The length of each obtained sequence was 735 bp.

Analysis performed in the BLAST sequence analysis tool revealed the similarity of obtained sequences to a number of metacestodes of *T. hydatigena* from southern Europe and Asia. Isolates from the liver of first wild boar and moose (GenBank accession nos: MF630923, MF630924) were identical to those obtained from sheep in Sardinia, Italy (GenBank accession nos: KT372528, KT372525, KT372524), Iran (GenBank accession no: JQ710593) and India (GenBank accession no: DQ995656). Metacestode from the liver of second wild boar could be compared to sequences of *T. hydatigena* from sheep in Italy (GenBank accession nos: KT372530, KT372529, KT372522) and Iran (GenBank accession no: JQ710588) as well as goats in Iraq (GenBank accession no: MN638348) and China (GenBank accession nos: KT258027, JN831298, JN831297, JN831295, JN831294, JN831291). Only the sequence of *T. hydatigena* metacestode from moose mediastinum had no matches in the BLAST analysis.

For more informative comparison, relevant sequences of *T. hydatigena* and other Taeniidae species were downloaded from the GenBank database and used in the Bayesian analysis (Table 1 in Appendix 1 in ESM).

**Figure 3:**
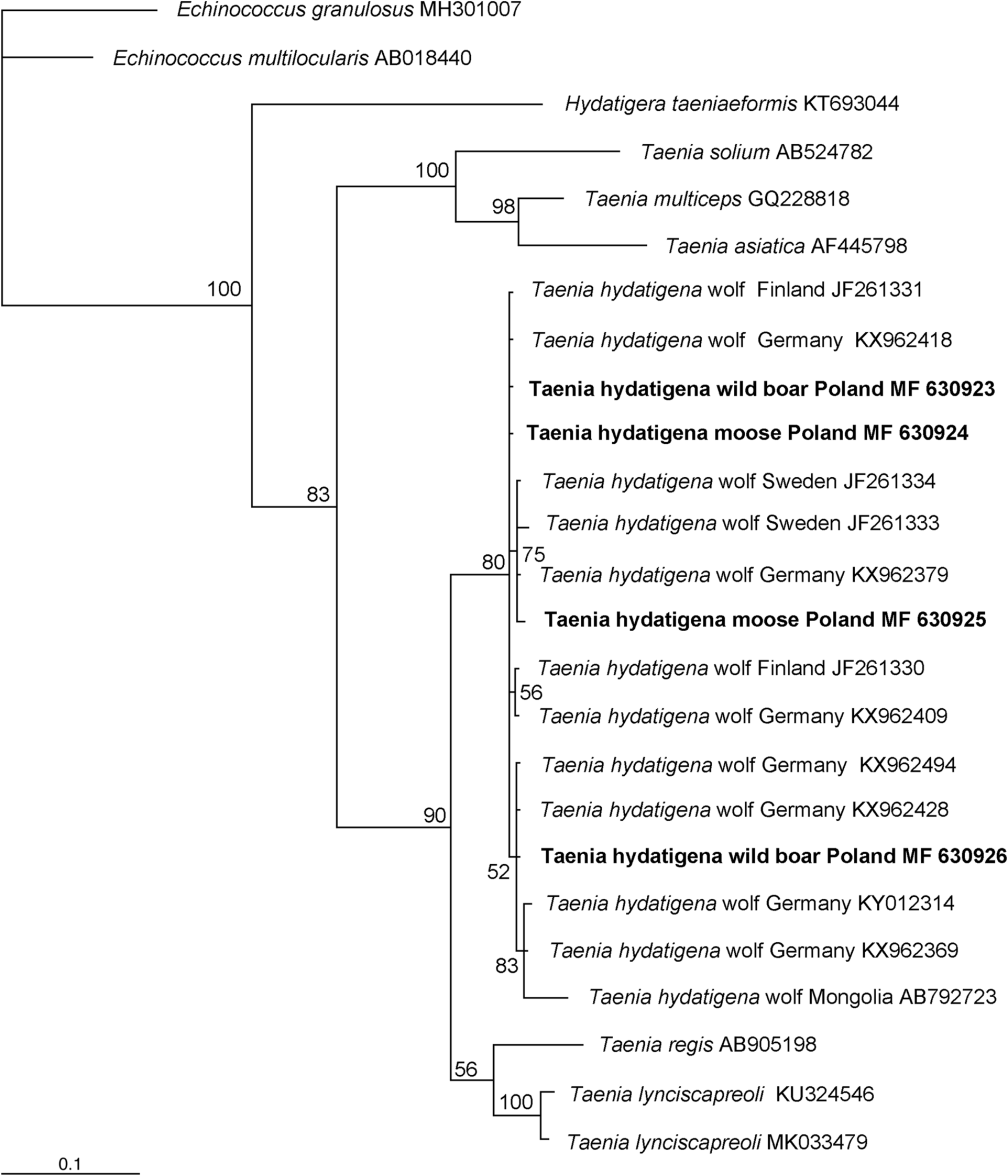
Phylogenetic tree of *Taenia hydatigena* haplotypes, constructed by Bayesian inference (BI) analysis using MrBayes version 3.2. For BI codon analysis (nucmodel = codon), the HKY + I + G model was chosen based on jModelTest version 2.1.4 (Guindon and Gascuel 2003; Darriba et al. 2012) using Akaike information criterion. Analysis was run for 3,000,000 generations, with 750,000 generations discarded as ‘burn-in’. Hosts, country and GenBank accession numbers of origin are shown (host and country are given only for *T. hydatigena* haplotypes). Nodal support is indicated as Bayesian posterior probabilities. Sequences from *Echinococcus granulosus* (MH301007), *E*. *multilocularis* (AB018440) and *Hydatigera taeniaeformis* (KT693044) were used as outgroup. Sequences generated in this study are shown in bold. The scale bars are proportional to the number of substitutions per site.

Bayesian analysis of *cox*1 sequences (Fig. 3) revealed that *T. hydatigena* from the liver of wild boar and moose (intermediate hosts) from Poland (GenBank accession nos.: MF630923, MF630924) are included in the subclade together with *T. hydatigena* from wolves (definitive host) in Germany (GenBank accession no: KX962418) and Finland (GenBank accession no: JF261331). *Taenia hydatigena* from the liver of second wild boar (GenBank accession no: MF630926) is included together with *T. hydatigena* from wolves in Germany (GenBank accession nos: KX962428, KX962494) whereas isolate of *T. hydatigena* from moose mediastinum (GenBank accession no. MF630925) is included in the subclade together with isolates from wolves in Sweden (GenBank accession nos. JF261333, JF261334) and Germany (GenBank accession no. KX962379).

Comparisons of both the nucleotide and amino acid sequences derived from the *cox*1 gene of the analyzed species of *Taenia* are presented in Table 2.

**Table 2:** Pairwise Comparison of Cytochrome *c* Subunit 1 Mitochondrial DNA Nucleotide Sequence and Inferred Amino Acid Sequence Variability Among 12 Selected Isolates of *Taenia* spp.

	1	2	3	4	5	6	7	8	9	10	11	12	13	14	15	16	17	18	19	20	21	22
1*. T. multiceps* GQ228818		3 (2,7)	4 (3,6)	4 (3,6)	4 (3,6)	4 (3,6)	4 (3,6)	4 (3,6)	4 (3,6)	4 (3,6)	4 (3,6)	4 (3,6)	4 (3,6)	4 (3,6)	10 (8,9)	4 (3,6)	4 (3,6)	4 (3,6)	4 (3,6)	5 (4,5)	5 (4,5)	4 (3,6)
2. *T. asiatica* AF445798	29 (8,6)		7 (6,2)	7 (6,2)	7 (6,2)	7 (6,2)	7 (6,2)	7 (6,2)	7 (6,2)	7 (6,2)	7 (6,2)	7 (6,2)	7 (6,2)	7 (6,2)	13 (11,6)	7 (6,2)	7 (6,2)	7 (6,2)	7 (6,2)	8 (7,1)	8 (7,1)	7 (6,2)
3. *T solium* AB524782	36 (10,7)	46 (13,7)		6 (5,4)	6 (5,4)	6 (5,4)	6 (5,4)	6 (5,4)	6 (5,4)	6 (5,4)	6 (5,4)	6 (5,4)	6 (5,4)	6 (5,4)	11 (9,8)	6 (5,4)	6 (5,4)	6 (5,4)	6 (5,4)	6 (5,4)	6 (5,4)	6 (5,4)
4. *T. hydatigena* wolf Sweden JF261334	43 (12,8)	45 (13,4)	49 (14,6)		0	0	0	0	0	0	0	0	0	0	6 (5,4)	0	0	0	0	1 (0,9)	1 (0,9)	1 (0,9)
5. *T. hydatigena* wolf Sweden JF261333	43 (12,8)	47 (14,0)	49 (14,6)	2 (0,6)		0	0	0	0	0	0	0	0	0	6 (5,4)	0	0	0	0	1 (0,9)	1 (0,9)	1 (0,9)
6. *T. hydatigena* wolf Finland JF261331	42 (12,5)	44 (13,1)	48 (14,3)	1 (0,3)	3 (0,9)		0	0	0	0	0	0	0	0	6 (5,4)	0	0	0	0	1 (0,9)	1 (0,9)	1 (0,9)
7. *T. hydatigena* wolf Finland JF261330	41 (12,2)	43 (12,8)	48 (14,3)	2 (0,6)	4 (1,2)	1 (0,3)		0	0	0	0	0	0	0	6 (5,4)	0	0	0	0	1 (0,9)	1 (0,9)	1 (0,9)
8. *T. hydatigena* wolf Germany KY012314	41 (12,2)	45 (13,4)	49 (14,6)	4 (1,2)	4 (1,2)	3 (0,9)	4 (1,2)		0	0	0	0	0	0	6 (5,4)	0	0	0	0	1 (0,9)	1 (0,9)	1 (0,9)
9*. T. hydatigena* wolf Germany KX962494	41 (12,2)	45 (13,4)	47 (14,0)	2 (0,6)	2 (0,6)	1 (0,3)	2 (0,6)	2 (0,6)		0	0	0	0	0	6 (5,4)	0	0	0	0	1 (0,9)	1 (0,9)	1 (0,9)
10. *T. hydatigena* wolf Germany KX962428	41 (12,2)	45 (13,4)	47 (14,0)	2 (0,6)	2 (0,6)	1 (0,3)	2 (0,6)	2 (0,6)	0		0	0	0	0	6 (5,4)	0	0	0	0	1 (0,9)	1 (0,9)	1 (0,9)
11. *T. hydatigena* wolf Germany KX962418	42 (12,5)	44 (13,1)	48 (14,3)	1 (0,3)	3 (0,9)	0	1 (0,3)	3 (0,9)	1 (0,3)	1 (0,3)		0	0	0	6 (5,4)	0	0	0	0	1 (0,9)	1 (0,9)	1 (0,9)
12. *T. hydatigena* wolf Germany KX962409	41 (12,2)	43 (12,8)	48 (14,3)	2 (0,6)	4 (1,2)	1 (0,3)	0	4 (1,2)	2 (0,6)	2 (0,6)	1 (0,6)		0	0	6 (5,4)	0	0	0	0	1 (0,9)	1 (0,9)	1 (0,9)
13*. T. hydatigena* wolf Germany KX962379	43 (12,8)	45 (13,4)	49 (14,6)	0	2 (0,6)	1 (0,3)	2 (0,6)	4 (1,2)	2 (0,6)	2 (0,6)	1 (0,6)	2 (0,6)		0	6 (5,4)	0	0	0	0	1 (0,9)	1 (0,9)	1 (0,9)
14. *T. hydatigena* wolf Germany KX962369	42 (12,5)	46 (13,7)	48 (14,3)	3 (0,9)	3 (0,9)	2 (0,6)	3 (0,9)	1 (0,3)	1 (0,3)	1 (0,3)	2 (0,6)	3 (0,9)	3 (0,9)		6 (5,4)	0	0	0	0	1 (0,9)	1 (0,9)	1 (0,9)
15. *T. hydatigena* wolf Mongolia AB792723	50 (14,9)	54 (16,1)	55 (16,4)	11 (3,3)	11 (3,3)	10 (3,0)	11 (3,3)	9 (2,7)	9 (0,9)	9 (2,7)	10 (3,0)	11 (3,3)	11 (3,3)	8 (2,4)		6 (5,4)	6 (5,4)	6 (5,4)	6 (5,4)	7 (6,2)	1 (0,9)	7 (6,2)
16. *T. hydatigena* wild boar Poland MF 630923	42 (12,5)	44 (13,1)	48 (14,3)	1 (0,3)	3 (0,3)	0	1 (0,3)	3 (0,9)	1 (0,3)	1 (0,3)	0	1 (0,3)	1 (0,3)	2 (0,6)	10 (3,0)		0	0	0	1 (0,9)	1 (0,9)	1 (0,9)
17*. T. hydatigena* moose Poland MF 630924	42 (12,5)	44 (13,1)	48 (14,3)	1 (0,3)	3 (0,9)	0	1 (0,3)	3 (0,9)	1 (0,3)	1 (0,3)	0	1 (0,3)	1 (0,3)	2 (0,6)	10 (3,0)	0		0	0	1 (0,9)	1 (0,9)	1 (0,9)
18*. T. hydatigena* moose Poland MF 630925	42 (12,5)	44 (13,1)	49 (14,6)	1 (0,3)	3 (0,9)	2 (0,6)	3 (0,9)	5 (1,5)	3 (0,9)	3 (0,9)	2 (0,6)	3 (0,9)	1 (0,3)	4 (1,2)	12 (3,6)	2 (0,6)	2 (0,6)		0	1 (0,9)	1 (0,9)	1 (0,9)
19. *T. hydatigena* wild boar Poland MF 630926	41 (12,2)	45 (13,4)	47 (14,0)	2 (0,6)	2 (0,6)	1 (0,3)	2 (0,6)	2 (0,6)	0	0	1 (0,6)	2 (0,6)	2 (0,6)	1 (0,3)	9 (2,7)	1 (0,3)	1 (0,3)	3 (0,9)		1 (0,9)	1 (0,9)	1 (0,9)
20. *T. lynciscapreoli* KU324546	45 (13,4)	45 (13,4)	47 (14,0)	23 (6,8)	25 (7,4)	22 (6,5)	21 (6,2)	23 (6,8)	23 (6,8)	23 (6,8)	22 (6,5)	21 (6,2)	23 (6,8)	24 (7,1)	31 (9,2)	22 (6,5)	22 (6,5)	23 (6,8)	23 (6,8)		1 (0,9)	2 (1,8)
21. *T. lynciscapreoli* MK033479	43 (12,8)	43 (12,8)	45 (13,4)	21 (6,2)	23 (6,8)	20 (5,9)	19 (5,6)	21 (6,2)	21 (6,2)	21 (6,2)	20 (5,9)	19 (5,6)	21 (6,2)	22 (6,5)	30 (8,9)	20 (5,9)	20 (5,9)	21 (6,2)	21 (6,2)	3 (0,9)		2 (1,8)
22*. T. regis* AB905198	47 (14,0)	46 (13,7)	50 (14,9)	28 (8,3)	28 (8,3)	27 (8,0)	26 (7,7)	28 (8,3)	26 (7,7)	26 (7,7)	27 (8,0)	26 (7,7)	28 (8,3)	27 (8,0)	35 (10,4)	27 (8,0)	27 (8,0)	28 (8,3)	26 (7,7)	23 (6,8)	22 (6,5)	

*Above diagonal* number of variable sites in 112 amino acids. *Below diagonal* number of variable sites in 336 base pairs. Percentage of variable sites between 2 isolates is given in parentheses

Nucleotide sequences of *T. hydatigena* from moose and wild boars diverged significantly from *T. multiceps* (GenBank accession no:GQ228818), *T. asiatica* (GenBank accession no:AF445798) and *T. solium* (GenBank accession no:AB524782) and ranged between 12.2 and 14.6%. Nucleotide sequence variation in the *cox*1 gene between metacestodes of *T. hydatigena* and isolates of *T. lynciscapreoli* (GenBank accession nos: KU324546) and *T. regis* (GenBank accession no:AB905198) was also significant (5.9–8.3%), whereas it reached only 0.9% at the amino acid level. The metacestodes isolated from the liver of wild boars and moose were identical at the amino acid level to those taken from wolves in Sweden (GenBank accession nos: JF261334, JF261333), Finland (GenBank accession nos:JF261331, JF261330) and Germany (GenBank accession nos.: KY012314, KX962494,KX962428, KX962418, KX962409, KX962379, KX962369), whereas their nucleotide sequences diverged slightly (0.30–1.5%).

In addition, greater divergence was found between the *cox*1 gene sequences present in our isolates and that obtained from Mongolian isolates (GenBank accession no. AB792723), ranging from 2.7 to 3.6%. Furthermore, European isolates of *T. hydatigena*, including those of the present study, and an isolate from a wolf from Mongolia also demonstrated significant differences at the amino acid level (5.4%).

Molecular and morphological identification confirmed the occurrence of *T. hydatigena* in the tested samples.

According to previous data, there is strong intraspecific variability of hook measures in *T. hydatigena* (Haukisalmi et al. 2011), which is confirmed in our study. There are several reports concerning measurements of *T. hydatigena* adult and metacestode stages (Verster 1969; Loos-Frank 2000; Gomez-Puerta et al. 2015; Singh et al. 2015). Size and shape of our metacestodes are consistent with the data given by Verster (1969) and Loos-Frank (2000). Large hooks of our metacestodes had higher total length than metacestodes from domestic ruminants in India with overlapping total length of small hooks (Singh et al. 2015), whereas large and small hooks of metacestodes from wild ruminants in Peru were smaller than ours (Gomez-Puerta et al. 2015). It might be a result of the effect of different intermediate host on rostellar hooks of metacestodes as well as environmental and genetic components (Lymbery 1998). In our studies, total and basal lengths of small hooks were higher in metacestodes from moose than from wild boars, which might be also a reflection of the intermediate host effect.

Although the intraspecific variability of *T. hydatigena* is considered to be high (Kędra et al. 2001), differences between our sequences of *T. hydatigena* metacestodes and *T. hydatigena* from European wolves were small and ranged from 0.30% to 1.5% (Table 2). The differences identified in nucleotide sequence between the European isolates of *T. hydatigena* were of the order of individual bases and did not affect their amino acid structure. In contrast, the amino acid sequence of *T. hydatigena* from Mongolia differed significantly from European isolates, which can be attributed to the geographical distribution of the tapeworm.

The genetic identity between the isolates from moose and wild boar analyzed in the present study and those taken from wolves from Western Europe indicate that wolves are definitive hosts of *T. hydatigena*. Recent studies have revealed that ungulates sharing the same territory with wolves tend to demonstrate a significantly higher prevalence of metacestode infection than ruminants inhabiting other areas (Lesniak et al. 2017). Therefore, infection of moose and wild boar in Poland with *T. hydatigena* may result from growing levels of wolf colonization across the country (Nowak and Mysłajek 2016). Recent studies indicate that the wolf population has increased from 500 to over 1500 individuals over the last decade (Diserens et al. 2017). The genetic similarity of our metacestodes to *T. hydatigena* from wolves across the German border may be accounted for by the fact that the wolves inhabiting north-eastern Poland migrated and settled in western Poland and Germany, forming a Central European wolf population (Czarnomska et al. 2013).

The rapid increase in the moose and wild boar populations in Poland (Filip and Demiaszkiewicz 2016; Flis 2011), together with the growing numbers of wolves, allow the life cycle of *T. hydatigena* to be easily closed. Although moose and wild boar are typical intermediate hosts of *T. hydatigena*, the parasite has not been commonly observed in wild mammals in Poland (Gadomska 1981; Tropiło and Kiszczak 1995). All previous studies in Poland referred to larvae of *T. hydatigena* located typically, in the abdominal cavity, and so it was in three out of four metacestodes isolated during this study. However, one of metacestodes from moose was found in the mediastinum. Radfar et al. (2005) observed *T. hydatigena* metacestodes in the thoracic cavity only in 1.24% of examined animals while most of data refer to larvae of *T. hydatigena* in the abdomen cavity (Järvis et al. 2007; Gomez-Puerta et al. 2015; Scala et al. 2015; Singh et al. 2015). The metacestode from moose mediastinum differs from other isolates not only by location in the host but also by different haplotype with no matches in the BLAST analysis. Detection of two different isolates of *T. hydatigena* metacestode in a single moose indicates two irrespective sources of the infection and can be evidence of a high level of contamination of the environment with invasive forms of the cestode. It might be a threat not only for domestic animals but also for farmed cervids, which are especially exposed to contact with both wild and domestic carnivores.

Although wolves, as definitive hosts of *T. hydatigena*, are considered as playing a marginal role in the spread of the cestode to domestic dogs, it is possible that hunting dogs fed with offal from ungulates or stray dogs fed with carrion may remain at serious risk of infection (Otranto et al. 2015). According to Lahmar et al. (2017), in some countries rural stray dogs are mainly transmitting the tapeworm in the environment. *Taenia hydatigena* was also detected in dogs in Poland and Ukraine (Okulewicz et al. 1994; Komyushin et al. 2013; Karamon et al. 2016). All infected animals came from rural areas, which can be evidence that dogs, on contact with wild animals and carrion, should be considered as an important source of *T. hydatigena* infection in this part of Europe. Also jakals, which have begun to expand in Poland recently may play a role in spreading *T. hydatigena* infection (Kowalczyk et al. 2015). Therefore, it is essential to begin regular monitoring of *T. hydatigena* infection in both wild and domestic animals in Poland and to determine which canids are involved in spreading the cestode in the wild.

The detection of *T. hydatigena* in wild mammals from eastern and central Poland and its similarity to *T. hydatigena* identified in wolves in Germany indicates that the recolonization of Western Europe by wild carnivores should be considered successful. However, the relationships between parasites, wild and domestic hosts, the size of the host population and its geographical distribution should be taken into account when estimating the risk of infection with parasitic diseases. Further studies are required to determine the prevalence of *T. hydatigena* in wild herbivores, omnivores and carnivores in Poland and to fully evaluate the risk of infection in domestic animals.

## Electronic Supplementary Material

Below is the link to the electronic supplementary material.

Supplementary material 1 (DOC 51 kb)
