# Supplementary material for: First Molecular Identification of Taenia hydatigena in Wild Ungulates in Poland
Source: Ecohealth. 2019 Jan 23;16(1):161–70. doi: 10.1007/s10393-019-01392-9 (PMC6430758; doi:10.1007/s10393-019-01392-9)
Supplement: Supplementary file 1 — Supplementary material 1 (DOC 51 kb) [file 10393_2019_1392_MOESM1_ESM.doc]

**Table 1.** List of taxa included in the molecular analysis using sequence data of cytochrome *c* oxidase subunit I (*cox*I). GenBank accession numbers MF630923, MF630924, MF630925, MF630926 are metacestodes of *Taenia hydatigena* isolated during this study. Localisation of larvae in the body of wild boars and moose is described in brackets.

* - definitive host, ^ - intermediate host

| **Species** | **Host** | **Region** | **GenBank** | **References** |
| --- | --- | --- | --- | --- |
| *Echinococcus granulosus* | *Homo sapiens*^ | Poland | MH301007 | Laurimäe et al., 2018 |
| *Echinococcus multilocularis* | *Clethrionomys rufocanus*^ | Japan | AB018440 | Nakao et al., 2002 |
| *Hydatigera taeniaeformis* | *Rattus norvegicus*^ | Cambodia | KT693044 | Lavikainen et al., 2016 |
| *Taenia multiceps* | *Canis lupus familiaris^*^* | China | GQ228818 | Jia et al., 2010 |
| *Taenia asiatica* | *Homo sapiens^*^* | Korea | AF445798 | Jeon and Eom, 2006 |
| *Taenia solium* | *Sus scrofa*^ | Nepal | AB524782 | - |
| *Taenia lynciscapreoli* | *Capreolus pygargus*^ | Russia | KU324546 | Haukisalmi et al., 2016 |
| *Taenia lynciscapreoli* | *Lynx lynx^*^* | Poland | MK033479 | - |
| *Taenia regis* | *Panthera leo^*^* | Kenya | AB905198 | Terefe et al., 2014 |
| *Taenia hydatigena* | *Canis lupus^*^* | Sweden | JF261334 | Lavikainen et al., 2011 |
| *Taenia hydatigena* | *Canis lupus^*^* | Sweden | JF261333 | Lavikainen et al., 2011 |
| *Taenia hydatigena* | *Canis lupus^*^* | Finland | JF261331 | Lavikainen et al., 2011 |
| *Taenia hydatigena* | *Canis lupus^*^* | Finland | JF261330 | Lavikainen et al., 2011 |
| *Taenia hydatigena* | *Canis lupus^*^* | Germany | KY012314 | Lesniak et al., 2017 |
| *Taenia hydatigena* | *Canis lupus^*^* | Germany | KX962494 | Lesniak et al., 2017 |
| *Taenia hydatigena* | *Canis lupus^*^* | Germany | KX962428 | Lesniak et al., 2017 |
| *Taenia hydatigena* | *Canis lupus^*^* | Germany | KX962418 | Lesniak et al., 2017 |
| *Taenia hydatigena* | *Canis lupus^*^* | Germany | KX962409 | Lesniak et al., 2017 |
| *Taenia hydatigena* | *Canis lupus^*^* | Germany | KX962379 | Lesniak et al., 2017 |
| *Taenia hydatigena* | *Canis lupus^*^* | Germany | KX962369 | Lesniak et al., 2017 |
| *Taenia hydatigena* | *Canis lupus^*^* | Mongolia | AB792723 | - |
| *Taenia hydatigena* | *Sus scrofa* (liver)^ | Poland | MF630923 |  |
| *Taenia hydatigena* | *Alces alces* (liver)^ | Poland | MF630924 |  |
| *Taenia hydatigena* | *Alces alces* (mediastinum) ^ | Poland | MF630925 |  |
| *Taenia hydatigena* | *Sus scrofa* (liver) ^ | Poland | MF630926 |  |

**References:**

Haukisalmi V, Konyaev S, Lavikainen A, Isomursu M, Nakao M (2016) Description and life-cycle of Taenia lynciscapreoli sp. n. (Cestoda, Cyclophyllidea). Zookeys 584:1-23; DOI:10.3897/zookeys.584.8171 [Online April 25, 2016]

Jeon HK, Eom KS (2006) Taenia asiatica and Taenia saginata: genetic divergence estimated from their mitochondrial genomes.Experimental Parasitology 113:58-61; DOI:10.1016/j.exppara.2005.11.018 [Online Match 20, 2006]

Jia WZ, Yan HB, Guo AJ, Zhu XQ, Wang YC, Shi WG, Chen HT, Zhan F, Zhang SH, Fu BQ, Littlewood DT, Cai XP (2010) Complete mitochondrial genomes of Taenia multiceps, T. hydatigena and T. pisiformis: additional molecular markers for a tapeworm genus of human and animal health significance.BMC Genomics 11:447; DOI:10.1186/1471-2164-11-447 [Online July 22, 2010]

Laurimäe T, Kinkar L, Romig T, Omer RA, Casulli A, Umhang G, Gasser RB, Jabbar A, Sharbatkhori M, Mirhendi H, Ponce-Gordo F, Lazzarini LE, Soriano SV, Varcasia A, Rostami Nejad M, Andresiuk V, Maravilla P, González LM, Dybicz M, Gawor J, Šarkūnas M, Šnábel V, Kuzmina T, Saarma U (2018) The benefits of analysing complete mitochondrial genomes: Deep insights into the phylogeny and population structure of Echinococcus granulosus sensu lato genotypes G6 and G7.Infection, Genetics and Evolution 64:85-94; DOI:10.1016/j.meegid.2018.06.016 [Online June 12, 2018]

Lavikainen A, Laaksonen S, Beckmen K, Oksanen A, Isomursu M, Meri S (2011) Molecular identification of Taenia spp. in wolves (Canis lupus), brown bears (Ursus arctos) and cervids from North Europe and Alaska. Parasitology International 60:289-295; DOI:10.1016/j.parint.2011.04.004 [Online May 6, 2011]

Lavikainen A, Iwaki T, Haukisalmi V, Konyaev SV, Casiraghi M, Dokuchaev NE, Galimberti A, Halajian A, Henttonen H, Ichikawa-Seki M, Itagaki T, Krivopalov AV, Meri S, Morand S, Näreaho A, Olsson GE, Ribas A, Terefe Y, Nakao M (2016) Reappraisal of Hydatigera taeniaeformis (Batsch, 1786) (Cestoda: Taeniidae) sensu lato with description of Hydatigera kamiyai n. sp. International Journal of Parasitology 46:361-374; DOI:10.1016/j.ijpara.2016.01.009 [Online March 5, 2016]

Lesniak I, Heckmann I, Heitlinger E, Szentiks CA, Nowak C, Harms V, et al. (2017) Population expansion and individual age affect endoparasite richness and diversity in a recolonising large carnivore population. Scientific Reports 7:41730; DOI: 10.1038/srep41730 [Online January 27, 2017]

Nakao M, Yokoyama N, Sako Y, Fukunaga M, Ito A (2002)The complete mitochondrial DNA sequence of the cestode Echinococcusmultilocularis (Cyclophyllidea: Taeniidae). Mitochondrion 1:497-509; DOI:10.1016/S1567-7249(02)00040-5 [Online June 5, 2002]

Terefe Y, Hailemariam Z, Menkir S, Nakao M, Lavikainen A, Haukisalmi V, Iwaki T, Okamoto M, Ito A (2014) Phylogenetic characterisation of Taenia tapeworms in spotted hyenas and reconsideration of the "Out of Africa" hypothesis of Taenia in humans. International Journal of Parasitology 44:533-541; DOI:10.1016/j.ijpara.2014.03.013 [Online May 9, 2014]
